# Radiation therapy for retroperitoneal sarcoma: practice patterns in North America

**DOI:** 10.1186/s13014-024-02407-8

**Published:** 2024-03-16

**Authors:** Samantha M. Ruff, Victor Heh, David J. Konieczkowski, Amblessed Onuma, Hayley M. Dunlop, Alex C. Kim, Valerie P. Grignol, Carlo M. Contreras, Timothy M. Pawlik, Raphael Pollock, Joal D. Beane

**Affiliations:** 1 Division of Surgical Oncology, Department of Surgery, The Ohio State University Wexner Medical Center and James Comprehensive Cancer Center, N924 Doan Hall, 410 W 10th Ave, Columbus, OH 43210 USA; 2 Department of Radiation Oncology, The Ohio State University Wexner Medical Center and James Comprehensive Cancer Center, Columbus, OH 43210 USA; 3 Department of Biomedical Informatics and Center for Biostatistics, The Ohio State University Wexner Medical Center, Columbus, OH 43210 USA

**Keywords:** Retroperitoneal sarcoma, Radiation therapy, Surgery

## Abstract

**Background:**

The addition of radiation therapy (RT) to surgery in retroperitoneal sarcoma (RPS) remains controversial. We examined practice patterns in the use of RT for patients with RPS over time in a large, national cohort.

**Methods:**

Patients in the National Cancer Database (2004–2017) who underwent resection of RPS were included. Trends over time for proportions were calculated using contingency tables with Cochran-Armitage Trend test.

**Results:**

Of 7,485 patients who underwent resection, 1,821 (24.3%) received RT (adjuvant: 59.9%, neoadjuvant: 40.1%). The use of RT decreased annually by < 1% (*p* = 0.0178). There was an average annual increase of neoadjuvant RT by 13% compared to an average annual decrease of adjuvant RT by 6% (*p* < 0.0001). Treatment at high-volume centers (OR 14.795, *p* < 0.0001) and tumor > 10 cm (OR 2.009, *p* = 0.001) were associated with neoadjuvant RT. In contrast liposarcomas (OR 0.574, *p* = 0.001) were associated with adjuvant RT. There was no statistically significant difference in overall survival between patients treated with surgery alone versus surgery and RT (*p* = 0.07).

**Conclusion:**

In the United States, the use of RT for RPS has decreased over time, with a shift towards neoadjuvant RT. However, a large percentage of patients are still receiving adjuvant RT and this mostly occurs at low-volume hospitals.

## Introduction

Retroperitoneal sarcomas (RPS) account for approximately 10–15% of all soft tissue sarcomas. There are over 100 different histologic types of sarcoma with leiomyosarcoma and liposarcoma being the most common in the retroperitoneum [1]. Surgical resection remains the cornerstone of treatment for patients with RPS, but recurrence rates are about 50% at 5 years. [2] Efforts to incorporate systemic therapies and/or radiation therapy (RT) into the multidisciplinary care of patients with RPS have had mixed results. Data on systemic therapies are limited, but is being prospectively investigated in the STRASS2 study [3]. On the other hand, given its benefits in the treatment of extremity soft tissue sarcoma, RT has been more thoroughly examined in relation to RPS as a means to improve local control and prevent recurrence. Additionally, in the neoadjuvant setting, RT may improve the likelihood of an R0 resection, control microscopic disease present beyond the surgical margins, and lessen the risk of intra-operative tumor cell dissemination. [4]

Multiple retrospective studies and meta-analyses suggest that neoadjuvant RT improves local control [1, 5–9]. More recently, a randomized phase III clinical trial demonstrated no effect of pre-operative RT on an investigator-defined composite endpoint of abdominal recurrence free survival [10]. However, post-hoc analyses demonstrated (1) a significant improvement in true local recurrence rates with pre-operative RT; (2) a potential loss of statistical power to detect abdominal recurrence free survival (ARFS) benefit due to poor RT protocol compliance; (3) a near-significant trend towards ARFS benefit for patients with well-differentiated liposarcomas and other low-grade histologies, a trend which achieved statistical significance on inclusion of an expansion cohort [11–13]. Given these controversies, this study set out to utilize a large national database to examine the practice patterns in the use of RT in patients with RPS over time and evaluate the factors associated with receiving neoadjuvant versus adjuvant RT.

## Methods

### Data source

This study is a retrospective review of the National Cancer Database (NCDB 2004–2017) and was designated as an exempt study for institutional review board approval. The NCDB is a joint project of the Commission on Cancer of the American College of Surgeons and the American Cancer Society. The NCDB is the source of the de-identified data used herein; the NCDB has not verified and are not responsible for the statistical validity of the data analysis or the conclusions derived by the authors.

### Study population

The NCDB database was queried for patients over the age of 18 with the following ICD-O-3 histology codes: 8800, 8801, 8802, 8803, 8804, 8805, 8810, 8811, 8815, 8830, 8840, 8850, 8851, 8852, 8853, 8854, 8855, 8857, 8858, 8890, 8891, 8894, 8895, 8896, 8933, 8935, 9040, 9041, 9043 who underwent resection between 2004 and 2017. All other histologies were excluded. Patients with known metastatic disease to the bone, liver, lung, or brain or coded for metastatic disease in the American Joint Committee on Cancer (AJCC) variable were excluded. In addition, any patient who had radiation therapy on the same day as surgery (therefore representing intraoperative radiation therapy, n = 9) or radiation therapy more than 90 days after surgery (possibly representing treatment for recurrent disease rather than adjuvant therapy, n = 219) were excluded.

### Study variables

Variables included in the final analysis included age, sex, race, ethnicity, great circle distance, insurance status, facility location, facility type, Charlson Deyo score, year of diagnosis, average annual hospital volume, tumor differentiation, histology type, tumor size, and tumor grade. As stated by the NCDB, demographic and tumor specific variables are recorded at the time of diagnosis. Race was categorized as: Caucasian, African American, Asian, Other (patients marked as other or American Indian descent), and unknown. Facility location was coded as either New England/East Coast (New England, middle Atlantic, and south Atlantic), Midwest (east north central and west north central), South (east south central and west south central), West Coast (mountain and pacific), and unknown. Breakdowns of states included in each of these categories can be found in the NCDB data dictionary. Annual hospital volume was calculated using the “facility key” variable using the same methods as Bagaria et al. [14] Histology type was grouped as leiomyosarcoma (codes 8890, 8891, 8896), liposarcoma (codes 8850, 8851, 8852, 8853, 8854, 8855, 8857, 8858), or other (codes 8800, 8801, 8802, 8803, 8804, 8805, 8810, 8811, 8815, 8830, 8840, 8894, 8895, 8933, 8935, 9040, 9041, 9043). Tumor size was changed from a continuous variable to a categorical variable by grouping patients into two categories: ≤ 10 cm or > 10 cm. The size 10 cm was chosen based on staging criteria (T1/T2 vs T3/T4).

### Statistical analysis

Quantitative data was summarized using mean (standard deviation) or median (interquartile quartile range [IQR]), whereas categorical data was summarized using sample size, n (percent (%)). To estimate the effect of covariates on binary outcomes, logistic regression with generalized estimating equations (GEE) methodology with robust standard errors was applied. This approach was preferred due to correlated nature of data with patients clustered in facilities. An important advantage of GEE is that it seeks to produce reliable estimates in the presence of many small clusters. We analyzed all data by categorizing missing values as unknown class in the multivariable model. Regression coefficients with 95% confidence intervals (CI) were exponentiated to derive odds ratios (OR) and 95% CI or expressed as (OR-1) × 100% to obtain percentage change. Model adequacy was established by use of model calibration and discrimination metrics. Statistical significance was determined at alpha = 0.05. SAS version 9.4 (2014, SAS Institute, Cary, NC).

## Results

### Demographics and tumor specific variables

A total of 5,664 patients underwent surgery alone (SA) without RT, 730 patients underwent neoadjuvant RT and surgery (NRT + S), and 1,091 underwent surgery and received adjuvant RT (ART + S). Patients had a mean age of 62.4 (± 13.2), 60.7 (± 12.6), and 60.9 (± 12.6) in the SA, NRT + S, and ART + S cohorts, respectively. Patients were predominately Caucasian in all three cohorts (SA: n = 4826, 85.2%, NRT + S: n = 621, 85.1%, ART + S: n = 899, 82.4%) and had a Charlson Deyo score of 0 (SA: n = 4329, 76.4%, NRT + S: n = 574, 78.6%, ART + S: n = 849, 77.8%). While the majority of patients were treated at academic medical centers (SA: n = 3324, 58.7%, NRT + S: n = 504, 69.0%, ART + S: n = 452, 41.4%), this did not necessarily correlate with high volume centers. Most patients were treated at centers that saw on average < 5 cases per year (SA: n = 4327, 76.4%, NRT + S: n = 498, 68.2%, ART + S: n = 1008, 92.4%). In addition, most patients had liposarcoma (SA: n = 3754, 66.3%, NRT + S: n = 392, 53.7%, ART + S: n = 534, 48.9%) and tumors > 10 cm (SA: n = 3390, 59.9%, NRT + S: n = 394, 53.9%, ART + S: n = 600, 54.9%). All demographic data can be found in Table 1.

**Table 1:** Comparison of no radiation, neoadjuvant radiation, and adjuvant radiation populations

	Surgery only N = 5664(%)*	Adjuvant radiation N = 1091 (%)*	Neoadj uvant radiation N = 730 (%)*	*p* value
Patient characteristics				
Age (Mean ± SD)	62.43 (13.19)	60.91 (12.59)	60.65 (12.57)	< 0.001
Sex				< 0.001
Male	2725 (48.1)	473 (43.4)	381 (52.2)	
Female	2939 (51.9)	618 (56.7)	349 (47.8)	
Race				0.022
Caucasian	4826 (85.2)	899 (82.4)	621 (85.1)	
Afican American	505 (8.9)	136 (12.5)	67 (9.2)	
Asian	174 (3.1)	37 (3.4)	23 (3.2)	
Other	99 (1.8)	10 (0.9)	11 (15)	
Unknown	60 (1.1)	9 (0.8)	8 (1.1)	
Ethnicity				< 0.001
No Hispanic	4973 (87.8)	952 (87.3)	675 (92.5)	
Hispanic	450 (7.9)	78 (7.2)	33 (4.5)	
Unknown	241 (4.3)	61 (5.6)	22 (3.0)	
Great Circle Distance (Median, IQR)	17.6 (6.9, 52.1)	11.7 (4.9, 28.2)	28.4 (9.8, 83.6)	< 0.001
Primary Payor				< 0.001
Medicaid, Medicare, Other Government Insurance	2742 (48.4)	520 (47.7)	333 (45.6)	
Private Insurance	2529 (44.7)	530 (48.6)	364 (49.9)	
No Insurance	173 (3.1)	30 (2.8)	22 (3.0)	
Unknown	220 (3.9)	11 (10)	11 (15)	
Facility Location				< 0.001
New England/East Coast	2291 (40.5)	403 (36.9)	364 (49.9)	
Mid-West	1327 (23.4)	301 (27.6)	173 (23.7)	
South	890 (15.7)	139 (127)	46 (6.3)	
West Coast	876 (15.5)	192 (17.6)	107 (14.7)	
Unknown	280 (4.9)	56 (5.1)	40 (5.5)	
Facility Type				< 0.001
Academic/Research Pro gram	3324 (58.7)	452 (41.4)	504 (69.0)	
Community Cancer Program	174 (3.1)	76 (6.9)	14 (1.9)	
Comprehensive Community Cancer Program	1314 (23.2)	373 (34.2)	106 (14.5)	
Integrated Network Cancer Program	572 (10.1)	134 (12.3)	66 (9.0)	
Unknown	280 (4.9)	56 (5.1)	40 (5.5)	
Charlson-DeyoScore				0.108
0	4329 (76.4)	849 (77.8)	574 (78.6)	
1	958 (16.9)	190 (174)	120 (16.4)	
2	266 (4.7)	34 (3.1)	21 (2.9)	
≥3	111 (19)	18 (17)	15 (2.1)	
Year of diagnosis				< 0.001
2004	268 (4.7)	82 (7.5)	21 (2.9)	
2005	320 (5.7)	76 (6.9)	14 (1.9)	
2006	320 (5.7)	79 (7.2)	33 (4.5)	
2007	371 (6.6)	99 (9.1)	28 (3.8)	
2008	384 (6.8)	76 (6.9)	33 (4.5)	
2009	354 (6.3)	88 (8.1)	41 (5.6)	
2010	361 (6.4)	90 (8.3)	51 (6.9)	
2011	443 (7.8)	91 (8.3)	59 (8.1)	
2012	430 (7.6)	91 (8.3)	52 (7.1)	
2013	415 (7.3)	82 (7.5)	66 (9.0)	
2014	477 (8.4)	83 (7.6)	66 (9.0)	
2015	526 (9.3)	56 (5.1)	68 (9.3)	
2016	500 (8.8)	48 (4.4)	87 (11.9)	
2017	495 (8.7)	50 (4.6)	111 (152)	
Annual hospital volume				< 0.001
Average < 5 cases per year	4327 (76.4)	1008 (92.4)	498 (68.2)	
Average 5–10 cases per year	662 (11.7)	74 (6.8)	113 (155)	
Average > 10 cases/year	675 (11.9)	9 (0.8)	119 (163)	
Tumor factors				
Tumor differentiation				< 0.001
Well differentiated	2010 (35.5)	199 (18.2)	166 (22.7)	
Moderately differentiated	731 (12.9)	173 (15.9)	92 (12.6)	
Poorly differentiated	1296 (22.9)	350 (32.1)	195 (26.7)	
Undifferentiated	865 (15.3)	187 (17.1)	140 (19.2)	
Unknown	762 (13.5)	182 (167)	137 (18.8)	
Histology				< 0.001
Leiomyosarcoma	1294 (22.9)	398 (36.5)	227 (31.1)	
Liposarcoma	3754 (66.3)	534 (48.9)	392 (53.7)	
Other	616 (10.9)	159 (14.6)	111 (15.2)	
Tumor size				< 0.001
< 10 cm	1143 (20.2)	374 (34.3)	133 (18.2)	
> 10 cm	3390 (59.9)	600 (54.9)	394 (53.9)	
Unknown	1131 (199)	117 (107)	203 (27.8)	
Tumor grade				< 0.001
Grade 1	1186 (20.9)	109 (9.9)	118 (16.2)	
Grade 2	642 (11.3)	127 (116)	100 (13.7)	
Grade 3	1323 (23.4)	314 (28.8)	268 (36.7)	
Unknown	2513 (44.4)	541 (49.6)	244 (33.4)	

*Rounded to nearest percentage

### Utilization of radiation therapy over time and survival analysis

**Fig. 1:**
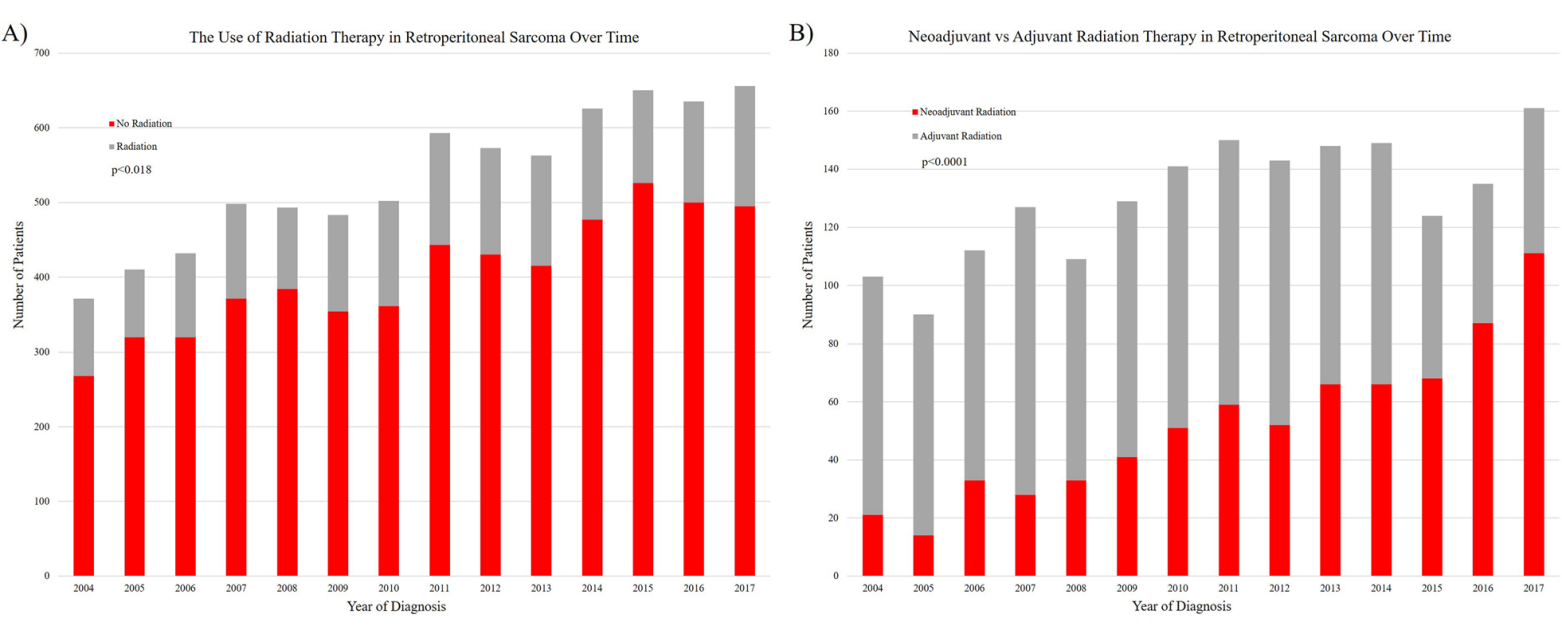
**A** The overall use of radiation therapy has decreased over time. **B** There has been a shift from using adjuvant radiation therapy to neoadjuvant radiation therapy

**Fig. 2:**
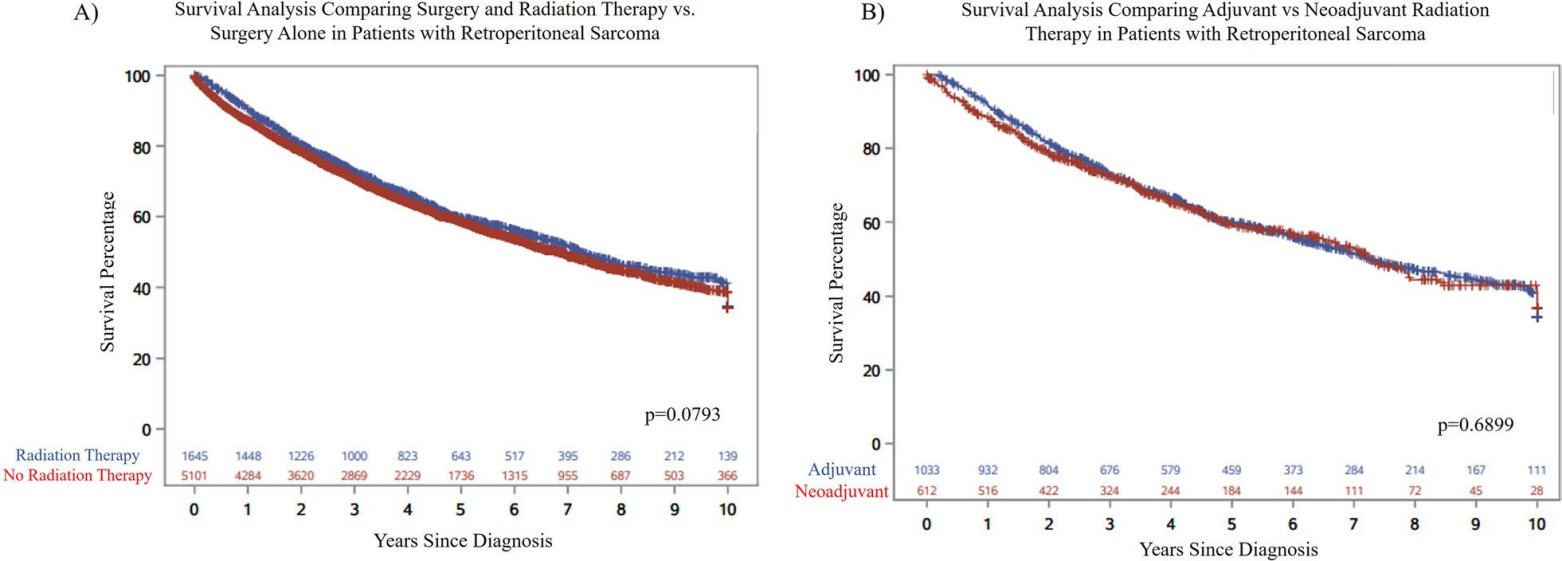
Survival analysis comparing. **A** Radiation therapy and surgery alone and **B** adjuvant and neoadjuvant therapy in patients with retroperitoneal sarcomas

Over time, the use of radiation therapy for the treatment of RPS decreased on average by < 1% per year (*p* = 0.018, Fig. 1A). Even though there was a decrease in RT, 24.5% of the population was still receiving RT in the most recent year of diagnosis (2017). There was also a shift from the use of adjuvant to neoadjuvant RT. On average, the use of neoadjuvant radiation therapy increased by 13% per year and the use of adjuvant radiation therapy decreased by 6% per year (*p* < 0.0001, Fig. 1B). There was no difference in overall survival comparing patients who received RT and surgery versus surgery alone (*p* = 0.0793, Fig. 2A). There was also no difference in overall survival among patients who received neoadjuvant versus adjuvant RT (*p* = 0.6899, Fig. 2B).

### Factors associated with receiving radiation therapy

On univariable analysis, age, sex, race, ethnicity, great circle distance, facility type, year of diagnosis, annual hospital volume, tumor differentiation, histology, tumor size, and tumor grade were each associated with receipt of RT and therefore, included in the multivariable analysis. On multivariable analysis, older patients (OR 0.984, CI 0.979–0.989, *p* < 0.0001) and patients treated at an academic/research program (OR 0.553, CI 0.384–0.797, *p* = 0.0015), comprehensive community cancer program (OR 0.648, CI 0.458–0.918, *p* = 0.0145), or integrated network cancer program (OR 0.577, CI 0.392–0.850, *p* = 0.0053) were less likely to receive radiation therapy compared to individuals treated at a community cancer program. In addition, patients with a later year of diagnosis were less likely to receive RT (OR 0.964, CI 0.933–0.997, *p* = 0.0308). Compared with patients who had well differentiated tumors, individuals with moderately differentiated tumors (OR 1.639, CI 1.1262–2.127, *p* = 0.0002), poorly differentiated tumors (OR 1.820, CI 1.451–2.283, *p* < 0.0001), or undifferentiated tumors (OR 1.665, CI 1.263–2.194, *p* = 0.0003) were associated with receipt of radiation therapy (Table 2). Patients with liposarcomas (OR 0.762, CI 0.615–0.857, *p* = 0.0002) compared to leiomyosarcoma and patients with larger tumors > 10 cm (OR 0.795, CI 0.684–0.923, *p* = 0.0026) were less likely to receive RT as shown in Table 3.

**Table 2:** Comparison of neoadjuvant radiation and adjuvant radiation populations

	Adjuvant Radiation N = 1091 (%)*	Neoadj uvant Radiation N = 730 (%)*	*p* value
Patient characteristics			
Age (Mean ± SD)	60.91 (12.59)	60.65 (12.57)	0.751
Sex			< 0.001
Male	473 (43.4)	381 (52.2)	
Female	618 (56.7)	349 (47.8)	
Race			0.174
Caucasian	899 (82.4)	621 (85.1)	
African American	136 (12.5)	67 (9.2)	
Asian	37 (3.4)	23 (3.2)	
Other	10 (0.9)	11 (15)	
Unknown	9 (0.8)	8 (1.1)	
Ethnicity			0.002
No Hispanic	952 (87.3)	675 (92.5)	
Hispanic	78 (7.2)	33 (4.5)	
Unknown	61 (5.6)	22 (3.0)	
Great Circle Distance (Median, IQR)	11.7 (4.9, 28.2)	28.4 (9.8, 83.6)	< 0.001
Primary Payor			0.672
Medicaid, Medicare, Other Government Insurance	520 (47.7)	333 (45.6)	
Private Insurance	530 (48.6)	364 (49.9)	
No Insurance	30 (2.8)	22 (3.0)	
Unknown	11 (10)	11 (15)	
Facility Location			< 0.001
New England/East Coast	403 (36.9)	364 (49.9)	
Mid-West	301 (27.6)	173 (23.7)	
South	139 (12.7)	46 (6.3)	
West Coast	192 (17.6)	107 (147)	
Unknown	56 (5.1)	40 (5.5)	
Facility Type			< 0.001
Academic/Research Pro gram	452 (41.4)	504 (69.0)	
Community Cancer Program	76 (6.9)	14 (1.9)	
Comprehensive Community Cancer Program	373 (34.2)	106 (14.5)	
Integrated Network Cancer Program	134 (12.3)	66 (9.0)	
Unknown	56 (5.1)	40 (5.5)	
Charlson-Deyo Score			0.859
0	849 (77.8)	574 (78.6)	
1	190 (17.4)	120 (164)	
2	34 (3.1)	21 (2.9)	
≥3	18 (1.7)	15 (2.1)	
Year of diagnosis			< 0.001
2004	82 (7.5)	21 (2.9)	
2005	76 (6.9)	14 (1.9)	
2006	79 (7.2)	33 (4.5)	
2007	99 (9.1)	28 (3.8)	
2008	76 (6.9)	33 (4.5)	
2009	88 (8.1)	41 (5.6)	
2010	90 (8.3)	51 (6.9)	
2011	91 (8.3)	59 (8.1)	
2012	91 (8.3)	52 (7.1)	
2013	82 (7.5)	66 (9.0)	
2014	83 (7.6)	66 (9.0)	
2015	56 (5.1)	68 (9.3)	
2016	48 (4.4)	87 (119)	
2017	50 (4.6)	111 (15.2)	
Annual hospital volume			< 0.001
Average < 5 cases per year	1008 (92.4)	498 (68.2)	
Average 5–10 cases per year	74 (6.8)	113 (15.5)	
Average > 10 cases/year	9 (0.8)	119 (16.3)	
Tumor Factors			
Tumor differentiation			0.008
Well differentiated	199 (18.2)	166 (22.7)	
Moderately differentiated	173 (15.9)	92 (12.6)	
Poorly differentiated	350 (32.1)	195 (26.7)	
Undifferentiated	187 (17.1)	140 (19.2)	
Unknown	182 (16.7)	137 (18.8)	
Histology			0.056
Leiomyosarcoma	398 (36.5)	227 (31.1)	
Liposarcoma	534 (48.9)	392 (53.7)	
Other	159 (14.6)	111 (152)	
Tumor size			< 0.001
< 10 cm	374 (34.3)	133 (18.2)	
> 10 cm	600 (54.9)	394 (53.9)	
Unknown	117 (107)	203 (27.8)	
Tumor Grade			< 0.001
Grade 1	109 (9.9)	118 (162)	
Grade 2	127 (11.6)	100 (137)	
Grade 3	314 (28.8)	268 (36.7)	
Unknown	541 (49.6)	244 (33.4)	

*Rounded to nearest percentage

**Table 3:** Multivariable analysis for factors associated with receiving any type of radiation therapy

	Odds ratio	Confidence interval	*p* value
Age	0.984	0.979–0.989	< 0.0001
Sex			
Female	Reference		
Male	1.101	0.979–1.24	NS
Race			
Caucasian	Reference		
African American	1.023	0.926–1.266	NS
Asian	1.135	0.772–1.668	NS
Other	0.671	0.360–1.250	NS
Unknown	0.818	0.379–1.769	NS
Ethnicity			
Hispanic	Reference		
Not Hispanic	1.378	1.027–1.849	0.0325
Unknown	1.454	0.912–2.318	NS
Great Circle Distance	1.000	0.999–1.000	NS
Facility Type			
Community Cancer Program	Reference		
Academic/Research Program	0.553	0.384–0.797	0.0015
Comprehensive Community Cancer Program	0.648	0.458–0.918	0.0145
Integrated Network Cancer Program	0.577	0.392–0.850	0.0053
Unknown	0.379	0.237–0.607	< 0.0001
Year of Diagnosis	0.964	0.933–0.997	0.0308
Annual Hospital Volume			
Average < 5 cases per year	Reference		
Average 5–10 cases per year	0.946	0.587–1.525	NS
Average > 10 cases/year	0.549	0.195–1.551	NS
Tumor differentiation			
Well differentiated	Reference		
Moderately differentiated	1.639	1.262–2.127	0.0002
Poorly differentiated	1.820	1.451–2.283	< 0.0001
Undifferentiated	1.665	1.263–2.194	0.0003
Unknown	1.812	1.423–2.308	< 0.0001
Histology			
Leiomyosarcoma	Reference		
Liposarcoma	0.726	0.615–0.857	0.0002
Other	0.887	0.706–1.113	NS
Tumor Size			
≤ 10 cm	Reference		
> 10 cm	0.795	0.684–0.923	0.0026
Tumor Grade			
Grade 1	Reference		
Grade 2	0.986	0.719–1.353	NS
Grade 3	1.301	0.992–1.705	NS
Unknown	0.907	0.688–1.196	NS

### Factors associated with receiving neoadjuvant radiation therapy

On univariable analysis, sex, ethnicity, great circle distance, facility type, year of diagnosis, annual hospital volume, tumor differentiation, tumor size, and tumor grade were all associated with receipt of neoadjuvant RT and included on the multivariable analysis (univariable analysis in Table 2). Histology was included on multivariable analysis for clinical significance. On multivariable analysis, male patients (OR 1.371, CI 1.066–1.762, *p* = 0.014) and traveling from further away (great circle distance OR 1.005, CI 1.001–1.010, *p* = 0.024) were associated with receiving neoadjuvant RT. Patients treated at academic/research programs (OR 6.162, CI 2.531–15.001, *p* < 0.0001) or integrated network cancer program (OR 4.261, CI 1.405–12.924, *p* = 0.011) compared to a community cancer program and treated at hospitals with an average of > 10 cases/year (OR 14.795, CI 6.058–36.136) compared to hospitals with < 5 cases/year were associated with receiving neoadjuvant RT over adjuvant RT. Patients with liposarcomas (OR 0.574, CI 0.409–0.805, *p* = 0.001) compared to leiomyosarcoma was associated with receiving adjuvant RT. Larger tumor size (> 10 cm, OR 2.009, CI 1.477–2.733, *p* < 0.0001) was associated with receiving neoadjuvant over adjuvant RT. The results of multivariable analysis are noted in Table 4.

**Table 4:** Multivariable analysis for factors associated with receiving neoadjuvant radiation therapy compared to adjuvant radiation therapy

	Odds ratio	Confidence interval	*p* value
Sex			
Female	Reference		
Male	1.371	1.066–1.762	0.014
Race			
Caucasian	Reference		
African American	0.752	0.493–1.146	NS
Asian	0.934	0.488–1.790	NS
Other	1.440	0.475–4.363	NS
Unknown	1.521	0.442–5.236	NS
Ethnicity			
Hispanic	Reference		
Not Hispanic	3.022	1.586–5.760	0.001
Unknown	2.188	0.892–5.366	NS
Great Circle Distance	1.005	1.001–1.010	0.024
Facility Type			
Community Cancer Program	Reference		
Academic/Research Program	6.162	2.531–15.001	< 0.0001
Comprehensive Community Cancer Program	2.345	0.976–5.631	NS
Integrated Network Cancer Program	4.261	1.405–12.924	0.011
Unknown	4.860	1.777–13.293	0.002
Year of Diagnosis	1.175	1.093–1.263	< 0.0001
Annual Hospital Volume			
Average < 5 cases per year	Reference		
Average 5–10 cases per year	1.762	0.773–4.015	NS
Average > 10 cases/year	14.795	6.058–36.136	< 0.0001
Tumor differentiation			
Well differentiated	Reference		
Moderately differentiated	0.827	0.479–1.427	NS
Poorly differentiated	0.487	0.317–0.749	NS
Undifferentiated	0.734	0.442–1.218	NS
Unknown	0.889	0.558–1.416	NS
Histology			
Leiomyosarcoma	Reference		
Liposarcoma	0.574	0.409–0.805	0.001
Other	0.842	0.563–1.259	NS
Tumor size			
≤ 10 cm	Reference		
> 10 cm	2.009	1.477–2.733	< 0.0001
Tumor Grade			
Grade 1	Reference		
Grade 2	0.758	0.398–1.443	NS
Grade 3	0.873	0.532–1.431	NS
Unknown	0.979	0.564–1.699	NS

## Discussion

The optimal role of RT in the treatment of RPS remains controversial. The NCDB is a valuable tool because it captures clinicopathologic, treatment, and survival data for patients with rare diseases that would otherwise be difficult to study. In view of that controversy, we found that approximately 25% of patients still receive RT for RPS. Additionally, even though we demonstrated a shift from adjuvant to neoadjuvant RT over this study period, approximately one third of patients who undergo RT receive it in the adjuvant setting.

The only curative approach to patients with RPS is complete surgical resection [15]. Oncologic outcomes following surgery are largely dependent on resection of all macro- and microscopic disease. Compared with extremity soft tissue sarcomas, an R0 resection can be more challenging in patients with RPS given anatomic constraints [16] While radical resection provides the only chance for long-term survival, approximately 50% of patients will develop a local recurrence [17]. As such, many centers continue to use RT in an attempt to improve recurrence free survival. The STRASS trial did not show a difference in abdominal recurrence free or overall survival in patients with RPS who received neoadjuvant RT and surgery compared to those treated with surgery alone [10]. However, subsequent efforts have underscored that true local recurrences were indeed reduced by almost half with the addition of preop RT; significant concerns regarding poor RT protocol compliance; and a trend towards ARFS benefit among low-grade histologies including well-differentiated liposarcomas, which in an expansion cohort ultimately achieved statistical significance [11–13]. Thus, some authorities continue to advocate for the role of radiotherapy in reducing local recurrences in appropriately selected patients.

When radiotherapy is employed for RPS, the sequence of radiation and surgery is well established. Adjuvant RT was previously explored for RPS, but is no longer recommended since dose-limiting critical structures, particularly bowel, often fill the surgical bed and cannot tolerate the doses of RT that are required in the post-operative setting [18, 19]. Correspondingly, adjuvant RT has been shown to increase postoperative complications, including intra-abdominal abscesses, hemorrhage, and bowel obstruction [20–22]. In the preoperative setting, toxicity is less as (1) the intact tumor typically displaces bowel and other organs at risk away from the radiation field, and (2) the lower dose of RT required in the pre-operative setting is far safer to these surrounding organs. Pre-operative RT may also induce fibrosis of the tumor’s capsule to help its detachment from neighboring organs [23]. Consistent with this shift in approach from post-operative to pre-operative RT, we found that a later year of diagnosis was associated with receiving neoadjuvant over adjuvant RT. Thus, at present, if RT is to be employed, pre-operative delivery is now the accepted standard of care. Of note, this situation differs markedly from extremity soft tissue sarcoma, where either pre- or post-operative approaches are typically viable (albeit with differing toxicity profiles). This emphasizes the need for multi-disciplinary evaluation of RPS patients in a planned fashion prior to surgery, rather than a reactive or reflexive referral for radiation in the event of unexpectedly adverse intra-operative and/or pathologic findings.

Given the controversy in the literature regarding the optimal role of pre-operative RT for RPS, attention is increasingly being focused on improving patient selection for RT. Zeh et al. utilized the NCDB to define prognostic factors among patients with RPS who received both RT and surgery [24]. These authors reported that in patients with AJCC stage 1 or 2 RPS, younger patients (< 61 years old) with good performance status and fibrosarcoma, well-differentiated liposarcoma, myxoid liposarcoma, and leiomyosarcoma had the best overall survival. Prior studies have shown mixed results regarding the effect of RT on overall survival. Our own study identified a trend (*p* = 0.07) towards improved overall survival among patients receiving pre-operative RT, but this did not reach statistical significance. Additionally, the strongest and most consistent evidence in support of a role of RT is for improvement of local control in RPS; however, we were not able to analyze this aspect as local control outcomes are not included in NCDB.

Histology is an additional factor in appropriate patient selection for RT [24]. Leiomyosarcoma is associated with a higher rate of distant rather than local recurrence while liposarcomas are associated with a higher rate of local recurrence [25, 26]. Since RT is aimed at decreasing local recurrence, this would suggest that liposarcomas may benefit more from neoadjuvant RT. Despite this, we found that leiomyosarcoma (compared to liposarcoma) was associated with receiving RT (compared to surgery alone) and neoadjuvant RT (compared to adjuvant RT), suggesting many patients may be receiving RT which is unlikely to achieve its stated aims. Additionally, our study found that tumor size (> 10 cm) was associated with receiving neoadjuvant RT, perhaps because the impressive size of these tumors facilitated consideration of multi-disciplinary care in a planned fashion prior to surgery.

When used in patients with RPS, RT should be in the neoadjuvant setting in appropriately selected patients. In this study, most patients were treated at low volume hospitals. Importantly, patients treated at a high-volume center (average > 10 cases/year) and/or at an academic program were more likely to receive neoadjuvant RT over adjuvant RT. Treatment at high volume centers has improved short and long term outcomes for many different cancer types and operations [27–30]. Consensus guidelines recommend that patients should be managed by a multidisciplinary team at specialized sarcoma centers that resect a minimum of 10–20 RPS per year. This team should include a surgeon, radiologist, pathologist, medical oncologist, and radiation oncologist with a concerted effort to contribute to prospective databases and enroll patients on clinical trials [15]. Patients with RPS treated at high-volume centers demonstrate better adherence to clinical practice guidelines, improved postoperative morbidity and mortality, and better overall survival [31–33].

Due to the retrospective nature of this study, there are a few limitations. First, as a large, national database, the NCDB has missing data from key variables and may have miscoded information. Second, the NCDB provides overall survival data, but does not provide data on recurrences. This is especially important in RPS since patients may have long term overall survival and treatment success of locoregional therapy (e.g. surgery, RT) is more commonly measured by recurrence rate and recurrence free survival in this disease. Despite these limitations, the large sample size of the NCDB still allows for important information to be gained, especially since the goal of this study was to evaluate the trends in RT over time.

In conclusion, the use of RT for RPS has decreased over time with a shift towards neoadjuvant radiation. Strikingly, however, many patients are still being treated in the adjuvant setting at low volume, community hospitals. When compared to surgery alone, we did not identify a statistically significant improvement in overall survival with the addition of RT. Taken together, patients with RPS need to be cared for by multidisciplinary teams at high-volume sarcoma referral centers where patients can be appropriately selected for neoadjuvant RT. Coordination between centers is crucial to accruing patients for phase III clinical trials to better define the use of RT in patients with distinct RPS histologies. Outreach programs to community hospitals with resources for treatment of RPS and improving access to tumor boards for multidisciplinary discussion may help provide an avenue to better standardizing care across North America.

## Data Availability

Not applicable.

## Abbreviations

RT: Radiation therapy
RPS: Retroperitoneal sarcoma
OR: Odds ratio
ARFS: Abdominal recurrence free survival
NCDB: National Cancer Database
AJCC: American Joint Committee on Cancer
IQR: Interquartile range
GEE: Generalized estimating equations
CI: Confidence interval
NRT + S: Neoadjuvant radiation therapy and surgery
ART + S: Adjuvant radiation therapy and surgery
SA: Surgery alone
